# Experimental Investigation into the Transmembrane Electrical Potential of the Forward Osmosis Membrane Process in Electrolyte Solutions

**DOI:** 10.3390/membranes4020275

**Published:** 2014-06-19

**Authors:** Lixia Bian, Yanyan Fang, Xiaolin Wang

**Affiliations:** Beijing Key Laboratory of Membrane Materials and Engineering, Department of Chemical Engineering, Tsinghua University, Beijing 100084, China; E-Mails: blx11@mails.tsinghua.edu.cn (L.B.); fyy04@mails.tsinghua.edu.cn (Y.F.)

**Keywords:** transmembrane electrical potential, forward osmosis, forward osmosis membrane, water flux, electrolyte

## Abstract

The transmembrane electrical potential (TMEP) in a forward osmosis membrane process with a single electrolyte solution as the draw and feed solutions was investigated by experiments. The effects of membrane orientation, the electrolyte species (KCl, NaCl, MgCl_2_, and CaCl_2_), concentration and concentration ratio of solutions at both sides of membrane on water flux and TMEP were investigated. The results showed that the TMEPs at different membrane orientation cannot completely coincide, which confirmed the effect of membrane asymmetry. The ion diffusion coefficients significantly affected the TMEP across the membrane, with different patterns for different electrolytes and concentrations.

## 1. Introduction

Forward osmosis (FO) is a process defined as the net movement of water across a semipermeable membrane toward a more concentrated solution. Because of the low hydraulic pressure required for this osmotic pressure driven (OPD) membrane process, FO possesses advantages of low energy consumption, low fouling tendency and high water recovery [1,2], and has attracted growing attention in seawater/brackish water desalination [3,4,5], wastewater treatment [6,7,8,9,10], liquid food processing [11,12,13], and power generation [14,15,16,17] via a derivative pressure-retarded osmosis process.

Most studies on the transport phenomenon in the FO process focus on the water and solute fluxes. The prior research reveals that most of the asymmetric structures of the FO membrane suffer from the drawback of internal concentration polarization (ICP) [18,19], which cannot be mitigated by altering hydrodynamic conditions such as increasing the flow rate or turbulence because it occurs within the membrane [20]. The effect of ICP on FO water flux has been modeled by adopting and modifying the classical solution-diffusion theory [19,21]. Reverse diffusion of the solute from the draw solution through the membrane to the feed solution is also inevitable because of the concentration difference. Recent studies have suggested careful consideration of reverse diffusion of the draw solute since it may be detrimental to the process [22], and have correlated the reverse transport of the draw solute to membrane fouling. These findings contribute to understanding the transport of water and solute molecules through the membrane in OPD membrane processes.

In the OPD membrane processes, electrolytes are usually used as the draw solute, and the transport of the electrolytes may be affected by the membrane electrical properties, which influences membrane performance, namely, flux and selectivity. Moreover, knowledge of the membrane properties, particularly the electrical properties, is required in the design and operation of the membrane process. However, the electrical properties and elecrokinetic phenomena of the FO membrane in OPD processes have been overlooked. Only one work has reported the zeta potentials of both sides of the FO membrane at different pH conditions, and the result indicates that the FO membrane is slightly negatively charged [23]. The charge properties of a membrane can be generally characterized by electrokinetic phenomena, including the zeta potential, membrane potential, streaming potential, and transmembrane electrical potential (TMEP), *etc.* Compared with other methods, the TMEP can be simultaneously measured with the mass transport, which makes it possible to actualize the online monitoring and diagnose membrane fouling. Therefore, the TMEP is an important electrokinetic phenomenon in hydraulic pressure driven (HPD) membrane processes such as nanofiltration (NF), and research on the TMEP promotes the understanding of the electrolytes transport mechanism in NF membranes [24,25,26].

In HPD membrane processes, the TMEP is the potential resulting from the convection of electrolyte solutions, diffusion of cations and anions, and difference between bulk and membrane phases, when a pressure gradient is applied through charged selective membranes. Analogously, the TMEP also exists across the membrane in FO membrane processes. Due to both sides of membrane do not exist hydraulic pressure difference in the FO membrane processes [27], the TMEP in FO membrane processes is different with that in HPD membrane processes. Although the operation mode of FO membrane process and the measurement of traditional membrane potential are almost identical, the TMEP in FO membrane process is significantly different from membrane potential, since the much higher water flux in FO membrane process would cause convection potential, which is not considered in the measurement of membrane potential due to the negligible water flow. Therefore, the TMEP in FO process consists of convection potential and membrane potential. Moreover, the TMEP can be simultaneously measured with the mass transport in the FO membrane process, thus it can reflect the relationship between the membrane performance and electrokinetic phenomena.

Notwithstanding the aforementioned advantages of TMEP, studies on the TMEP across membranes in the FO process are still limited. Therefore, it is quite necessary and very important to investigate and discuss the factors influencing the TMEP in FO membrane process.

In this study, the TMEPs of a FO membrane in four electrolyte solutions (KCl, NaCl, MgCl_2_, and CaCl_2_) are studied by experiments. The effects of membrane orientation, the concentration ratio of solutions in both sides of membrane and the electrolyte species on water flux and TEMP are investigated. This work is the first step to investigate the experimental phenomena of TMEP, which would raise the understanding of TMEP and provide basis experimental data for further analysis of TMEP in FO membrane process.

## 2. Results and Discussion

### 2.1. Influence of Membrane Orientation

**Figure 1:**
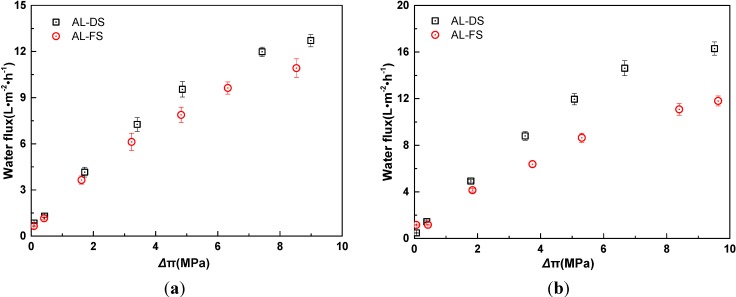
Relationship of water flux and osmotic pressure difference in two membrane orientations for KCl as draw solute (*t* = 25 °C). AL-DS: active layer facing the draw solution; AL-FS: active layer facing the feed solution. (**a**) *C*_d_/*C*_f_ = 8; (**b**) *C*_f_ = 0.01 M. The osmotic pressure difference refers to the effective values after correction for the effect of external concentration polarization (ECP).

Figure 1 shows the relationship between water flux and osmotic pressure difference in two membrane orientations. In this work, the osmotic pressure is calculated by commercial software Aspen plus. The effect of external concentration polarization on the osmotic pressure has been eliminated. As indicated in Figure 1, water fluxes increase with the increase of the osmotic pressure difference in both membrane orientations, and water fluxes in AL-DS (active layer face the draw solution) mode are higher than that in AL-FS (active layer face the feed solution) mode. With increased osmotic pressure difference, the driving force rises, which results in enhancement of water flux. When active layer faces the feed solution, more serious ICP will occur in high concentration of KCl, which leads to lower water flux [19].

Due to the diffusion coefficients of K^+^ and Cl^−^ in the bulk are almost equal, and the ionic radius and the ionic transport numbers of these two ions are close. When the diffusion coefficient of cation and anion is equal, the analytic expression would be simpler in some cases, which is conductive to understand some phenomena and laws.

**Figure 2:**
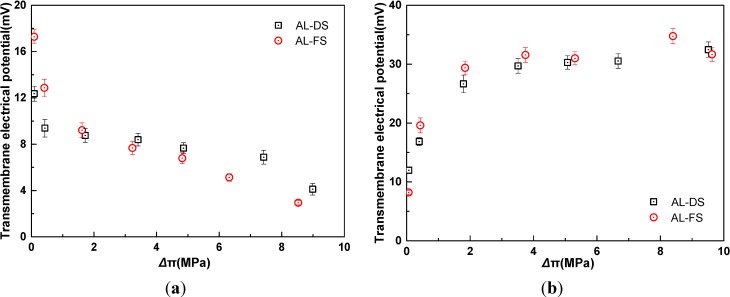
Relationship of transmembrane electrical potential and osmotic pressure difference in two membrane orientations for KCl as draw solute (*t* = 25 °C). AL-DS: active layer facing the draw solution; AL-FS: active layer facing the feed solution. (**a**) *C*_d_/*C*_f_ = 8; (**b**) *C*_f_ = 0.01 M. The osmotic pressure difference refers to the effective values after correction for the effect of ECP.

As mentioned above, the TMEP in FO process consists of convection potential and membrane potential. The convection potential is caused by the flux of electrolytes in the charged pores coupled with water flux under the osmotic pressure gradient. The membrane potential is composed of Donnan potential and diffusion potential. The Donnan potential is the electrical potential at the interfaces (feed-membrane and membrane-draw) resulted from the adsorption difference of cations and anions. The diffusion potential establishes by the imbalance of anion and cation concentrations, which is caused by the different mobility between anion and cation in the diffusion process from the high-concentration side to the low-concentration side in FO membrane. Due to the direction of water flow being opposite to that of ions diffusion, the sign of convection potential is opposite to that of diffusion potential. In Figure 2a, when the concentration ratio of the both sides of the membrane is *C*_d_/*C*_f_ = 8, the TMEP declines with the increase of osmotic pressure difference in two modes. This is due to the fact that the increased concentrations of draw and feed solution are in fixed ratio, which makes the KCl solution activity ratio in the both sides of the membrane remains almost constant. Meanwhile, diffusion coefficients of K^+^ and Cl^−^ are approximately equal, thus the diffusion potential is insignificant and keeps mostly unchangeable. On the other hand, with the increased solution concentration, the membrane charge is screened by the counter ion, the electrical double layers formed in the pores are compressed and hence the entire pore volume is electrically neutral, then the Donnan potential difference is decrescent. The Donnan potential predominates in TMEP in the low concentration, thus the TMEP decreases in two modes. Moreover, the convection potential enhances with the increased water flux, which contributes a little to the decrease of the TMEP due to the sign of the convection potential is contrary to that of membrane potential. The TMEPs at different membrane orientations cannot completely coincide, which confirms the effect of membrane asymmetry.

Figure 2b shows that membrane orientation affects TMEP at low concentrations of KCl, while as the concentration increases, the effect of membrane orientation on TMEP can be neglected. When the feed solution concentration is fixed at 0.01 M, at low concentration of draw solution, Donnan potential is dominant in TMEP. As the concentration of draw solution increases, Donnan potential in the side of draw solution becomes lower while that in the side of feed solution is constant, so the TMEP increases. When the draw solution concentration increases, namely, the concentration ratio of the draw and feed solution enhances, Donnan potential becomes insignificant compared with the diffusion potential. As the concentration increase, convection potential and diffusion potential are not affected by membrane orientation. Therefore, the effect of membrane orientation on TMEP becomes unimportant.

### 2.2. Influence of Solution Concentration Ratio

**Figure 3:**
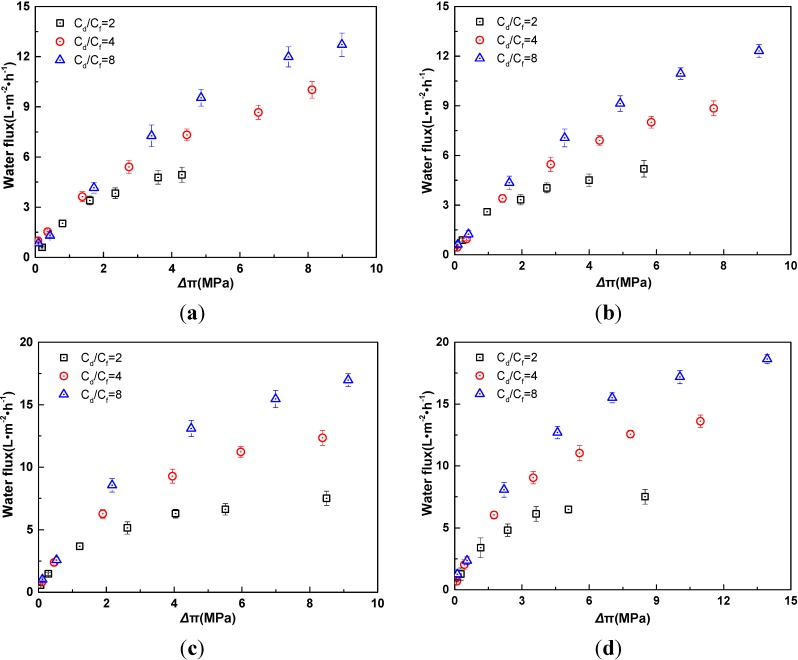
Relationship of water flux and osmotic pressure difference with different ratio of feed concentration and draw concentration in AL-DS mode (*t* = 25 °C). (**a**) KCl; (**b**) NaCl; (**c**) MgCl_2_; (**d**) CaCl_2_. The osmotic pressure difference refers to the effective values after correction for the effect of ECP.

As shown in Figure 3, water fluxes for four electrolytes enhance with the increase of osmotic pressure difference and have the same trend with the increase of the concentration ratio between draw solution and feed solution. At the same osmotic pressure difference, the decrease of the concentration ratio would lead to more serious ICP, and then results in a lower water flux.

**Figure 4:**
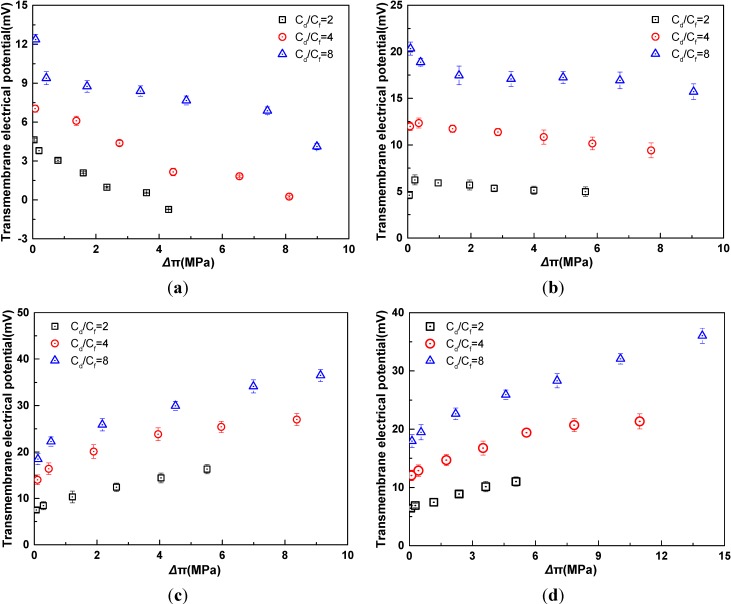
Relationship of transmembrane electrical potential and osmotic pressure difference with different ratio of feed concentration and draw concentration in AL-DS mode(*t* = 25 °C). (**a**) KCl; (**b**) NaCl; (**c**) MgCl_2_; (**d**) CaCl_2_. The osmotic pressure difference refers to the effective values after correction for the effect of ECP.

In the experiment, the concentration ratio increases by the way that the feed solution concentration decreases while the draw solution concentration is fixed. As indicated in Figure 4, with the increased the concentration ratio, the TMEP increases for four electrolytes. The reason for this phenomenon is that with the increased concentration ratio, the feed concentration decreases, leading to the increase of the diffusion potential and Donnan potential, while water flux increases leading to the increase of convection potential. The increment of the membrane potential is higher than that of the convection potential. Therefore, TMEP increases with the increase of concentration ratio for four electrolytes.

### 2.3. Influence of Electrolyte Species

**Figure 5:**
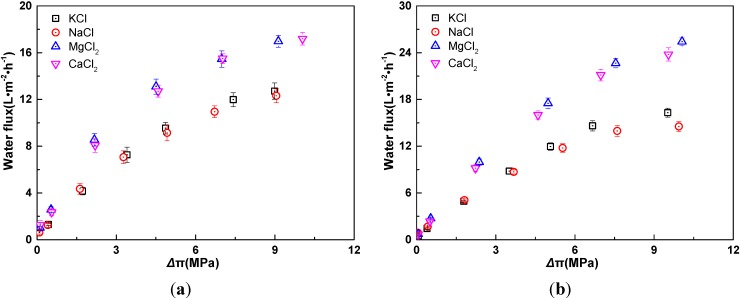
Relationship of water flux and effective osmotic pressure difference for four draw solutes in AL-DS mode (*t* = 25 °C). (**a**) *C*_d_/*C*_f_ = 8; (**b**) *C*_f_ = 0.01 M. The osmotic pressure difference refers to the effective values after correction for the effect of ECP.

The relationship between water flux and effective osmotic pressure difference for four draw solutes is shown in Figure 5. With the increase of effective osmotic pressure difference, water flux enhances. At the same effective osmotic pressure difference, water flux generated by bivalent salts is higher than that generated by univalent salts.

**Figure 6:**
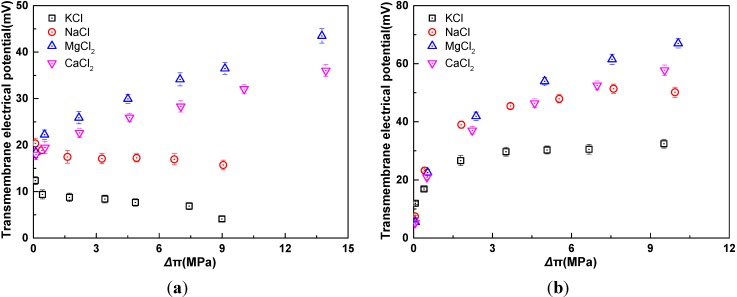
Relationship of transmembrane electrical potential and osmotic pressure difference for four draw solute in AL-DS mode (*t* = 25 °C). (**a**) *C*_d_/*C*_f_ = 8; (**b**) *C*_f_ = 0.01 M. The osmotic pressure difference refers to the effective values after correction for the effect of ECP.

As indicated in Figure 6, the electrolyte species have significant effect on TMEP. The diffusion coefficients of K^+^, Na^+^, Mg^2+^, Ca^2+^, and Cl^−^ are 1.96 × 10^−9^ m^2^/s, 1.33 × 10^−9^ m^2^/s, 0.705 × 10^−9^ m^2^/s, 0.793 × 10^−9^ m^2^/s, and 2.03 × 10^−9^ m^2^/s, respectively [28]. Therefore, the order of ions diffusion coefficient is Mg^2+^ < Ca^2+^ < Na^+^ < K^+^ < Cl^−^. The more the difference of diffusion coefficients between the cation and anion is, the more diffusion potential would be, so the order of diffusion potential is MgCl_2_ > CaCl_2_ > NaCl > KCl. On the other hand, the water flux generated by MgCl_2_ and CaCl_2_ solutions is relatively larger than that by KCl and NaCl solutions, which leads to the larger convection potential. However, the increment of the convection potential is lower than that of the diffusion potential. Moreover, as the concentration of electrolytes rises, Donnan potential becomes low and contributes little to the TMEP. Therefore, at the same osmotic pressure difference, the TMEP decreases in the following order: MgCl_2_ > CaCl_2_ > NaCl > KCl. In Figure 6a, another interesting phenomenon is that the TMEPs of univalent and bivalent salts show diverging trends. For univalent salts, the TMEPs decrease with the increase of effective osmotic pressure difference. Conversely, for bivalent salts, the TMEPs increase with the increase of effective osmotic pressure difference. This phenomenon can be interpreted by the following explanation.

In FO process, the direction of water flow is opposite to that of ions diffusion. The sign of convection potential is contrary to that of diffusion potential. Moreover, the convection potential has a positive correlation with water flux. The diffusion potential is related to the difference of diffusion coefficients between anion and cation as well as the activity ratio between draw solution and feed solution [29].

For univalent salts, the activity coefficients change a little at high concentration [30]. Thus, when *C*_d_/*C*_f_ is fixed at 8, the activity coefficient ratio of draw and feed solution is close to 8, which results in little variation of the diffusion potential varies a little. In spite of little variation of is fixed, with the salt concentrations of the solution in both sides of membrane increase, the concentration difference between the draw solution and feed solution enlarges, which leads to the increase of water flux, thus the convection potential increases. The sign of the convection potential is contrary to that of the diffusion potential. Therefore, the TMEPs decrease slightly with the increase of the osmotic pressure difference.

For bivalent salts, the activity coefficients change sharply at high concentration [31]. Therefore, when *C*_d_/*C*_f_ is fixed at 8, the activity coefficient ratio draw and feed solution increases quickly with the concentration increases at high concentration. This results in the increase of the activity ratio of draw and feed solution, leading to increased diffusion potential. This increase is exceptional large that the TMEPs increases with increased osmotic pressure difference, although the contrary convection potential increases with the increased water flux. Moreover, the detailed explanation for this phenomenon should be further investigated in the subsequent studies.

## 3. Experimental Section

### 3.1. Membrane and Chemicals

The FO membrane used in this work was provided by Hydration Technologies Innovations (Albany, OR, USA). This membrane is thought to be made of cellulose triacetate. The thickness of the membrane is less than 50 μm and its structure is quite different from standard RO membranes. RO membranes typically consist of a very thin active layer and a thick porous support layer. However, in the HTI FO membrane, the mechanical support is provided by an embedded polyester mesh instead of the thick support layer. Details on the FO membrane have been described elsewhere [1,2].

Four electrolyte solutions were used, including KCl, NaCl, MgCl_2_ and CaCl_2_ (reagent grade, Beijing Modern Eastern Fine Chemical Corporation, Beijing, China). All solutions were prepared using deionized water, and the concentrations were measured by the method of inductively coupled plasma-atomic emission spectrometry (iCAP 6300, ThermoFisher, Waltham, MA, USA).

### 3.2. Water Flux and TMEP Measurements

**Figure 7:**
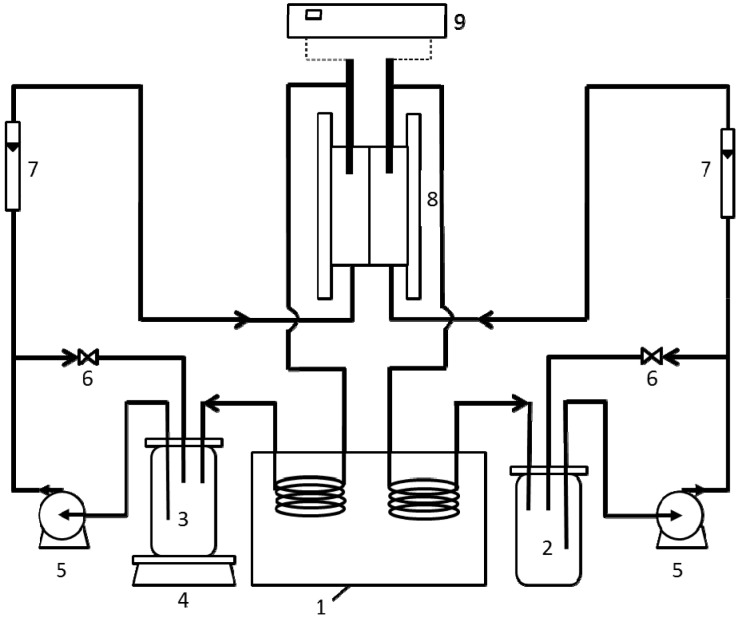
Schematic diagram of FO setup: (1) water bath; (2) draw solution; (3) feed solution; (4) electronic balance; (5) diaphragm pump; (6) valve; (7) flow meter; (8) membrane module; (9) multimeter.

The water flux and TMEP were measured synchronously in a laboratory setup as shown in Figure 7. The draw and feed solutions were pumped concurrently in each channel on both sides of the membrane in closed loops. The electrical potential difference was measured by two Ag/AgCl electrodes. The TMEP was obtained by subtracting the electrode potential from the potential directly read on multimeter. An electrical balance was used to monitor the weight reductions of the feed solution due to water transport through the membrane from the feed solution to the draw solution, from which water volumetric flux *J*_w_ was calculated. In all experiments, the temperature of the solutions maintained at 25.0 ± 0.2 °C. The tangential flow rate was fixed at 2.0 L∙min^−1^. Under these operation conditions and corresponding membrane module dimensions, the effect of ECP in our experiments can be neglected. However, in order to obtain accurate results, we still use the method presented by McCutcheon and Elimelech [32] to correct the influence of ECP.

## 4. Conclusions

In this work, the TMEPs of a FO membrane in four electrolyte solutions (KCl, NaCl, MgCl_2_, and CaCl_2_) are studied by experiments. The effects of membrane orientation, the concentration ratio of solutions in both sides of membrane and the electrolyte species on water flux and TEMP are investigated.

The water flux in AL-FS mode is lower than that in AL-DS mode owing to the effect of ICP. With the increase of concentration ratio, water flux increases due to the alleviation of ICP. Moreover, because they draw solute, water fluxes generated by bivalent salts are higher than that generated by univalent salts.

The TMEPs under different membrane orientations cannot completely coincide, which confirms the effect of membrane asymmetry on TMEP. The TMEP increases with increased concentration ratio of feed and draw solutions. Electrolyte species and the diffusion coefficients of ions have remarkable effects on TMEPs. The greater the difference of diffusion coefficients between the cation and anion is, the more TMEP would be for all four electrolytes. With the increase of effective osmotic pressure difference, the TMEPs of univalent and bivalent salts show diverging trends. This work is a step for the investigation of TMEP in FO processes and only indicates corresponding basic experimental results. The other factors regarding TMEP could be further studied.
